# Evaluation of *EPAS1* variants for association with bovine congestive heart failure

**DOI:** 10.12688/f1000research.19951.1

**Published:** 2019-07-25

**Authors:** Michael P. Heaton, Adam S. Bassett, Katherine J. Whitman, Greta M. Krafsur, Sang In Lee, Jaden M. Carlson, Halden J. Clark, Helen R. Smith, Madeline C. Pelster, Veronica Basnayake, Dale M. Grotelueschen, Brian L. Vander Ley

**Affiliations:** 1 U.S. Meat Animal Research Center, Clay Center, Nebraska, 68933, USA; 2 University of Nebraska-Lincoln, Clay Center, Nebraska, 68933, USA; 3 Anschutz School of Medicine, University of Colorado Denver, Aurora, Colorado, 80045, USA; 4 Neogen GeneSeek Operations, Lincoln, Nebraska, 68504, USA

**Keywords:** Heart failure, cattle, EPAS1, HIF2A, brisket disease, pulmonary hypertension, feedlot

## Abstract

**Background:**  Bovine congestive heart failure (BCHF) has become increasingly prevalent in feedlot cattle in the Western Great Plains of North America. BCHF is an untreatable complex condition involving pulmonary hypertension that culminates in right ventricular failure and death. A protein variant of hypoxia-inducible factor 2 alpha (HIF2α, encoded by the endothelial PAS domain-containing protein 1 gene, *EPAS1*) was previously reported to be associated with pulmonary hypertension at altitudes exceeding 2,000 m. Our aim was to evaluate *EPAS1* haplotypes for association with BCHF in feedlot cattle raised at moderate altitudes (1,200 m).

**Methods:** Paired samples of clinical cases and unaffected controls were collected at four feedlots in Nebraska and Wyoming. Each pair (n =102) was matched for source, pen, breed type, sex, arrival date, and management conditions. Cases were identified by animal caretakers, euthanized, and diagnosis was confirmed at necropsy. Cases were derived from 30 different ranch operations, with the largest source contributing 32. Animals were tested for eight *EPAS1* haplotypes encoding 36 possible different diploid combinations.

**Results:** The common, ancestral *EPAS1* haplotype encoding HIF2α with alanine (A) at position 606 and glycine (G) at position 610 was equally frequent in cases and controls (0.67). The *EPAS1* variant haplotype reported to be associated with disease (encoding threonine (T) at position 606 and serine (S) at position 610) was not enriched in cases compared with controls (0.21 and 0.25, respectively). Frequencies of other *EPAS1* haplotypes (e.g., encoding Q270, L362, or G671) were each less than 0.05 overall. McNemar’s test with 45 discordant pairs showed the linked T606/S610 variant was not associated with BCHF (OR = 0.73, CI _95_ 0.38 -1.4, *p*-value = 0.37).

**Conclusions:** HIF2α polypeptide variants were not significantly associated with BCHF in feedlot cattle at moderate altitudes. Thus, a wider search is needed to identify genetic risk factors underlying this disease.

## Introduction

Brisket disease has been known in the Rocky Mountain Region of Colorado and Utah for more than 100 years as a high altitude disorder characterized by severe ventral edema of the chest tissues ^1, 2^. In affected cattle, the reduced partial pressure of oxygen at high altitudes causes pulmonary hypoxia, vascular resistance, arterial remodeling, and pulmonary hypertension. As late as 1963, reports of brisket disease were limited to cattle grazed at altitudes greater than 2,100 m, with a prevalence of 2–10% ^3^. This condition, attributed to ‘cor pulmonale’, eventually causes right ventricular overload and enlargement, ultimately leading to heart failure. In 1976, a similar condition was reported in yearling feedlot cattle maintained at a lower altitude of 1,600 m ^4^. Since the 1970’s, bovine congestive heart failure (BCHF) has become increasingly common in feedlot cattle maintained at the low to moderate altitudes of the North American Plains (800 to 1,600 m) ^5^. However, it is uncertain whether hypobaric hypoxia is the underlying cause of BCHF in these cattle, and evidence suggests that left heart dysfunction may initiate BCHF ^6^. Histopathological assessment of cardiopulmonary tissues obtained from affected cattle fattened at 544-1,420 m revealed significant ventricular fibrosis, abundant cardiac adipose depots, coronary artery injury, and pulmonary venous remodeling ^6^. These features were phenotypically distinct from those of cattle with cor pulmonale at high altitudes. However, other evidence in similarly affected cattle (1,369 m) suggested death occurred prior to the development of advanced obesity ^7^. Thus, disease pathogenesis of BCHF in feedlot cattle maintained at the moderate altitudes remains unclear.

The impact of BCHF on animal mortality is substantial and appears to be increasing. Mortality in the U.S. and Canada has been estimated at 11 per 10,000 animals entering feedlots, with the rate doubling from 2002 to 2012 ^5^. Personal communication between authors (MPH and BLVL) and a Nebraska feedlot owner (G. Darnall) in 2018 indicated a BCHF prevalence as high as 7.5% in some single source lots of cattle. Affected cattle were typically bred and managed with the aim of achieving high carcass quality. For some affected producers in the Western Plains, BCHF is their single most costly health-related problem, with losses exceeding $250,000 annually in individual operations, surpassing those from bovine respiratory disease. Consequently, reducing the impact of BCHF is a high priority for the cattle industry.

A potential genetic risk factor for BCHF was reported in 2015 for cattle with high-altitude pulmonary hypertension (1,478 to 2,618 m) ^8^. Angus cattle affected with pulmonary hypertension had a higher frequency of the endothelial PAS domain-containing protein 1 gene ( *EPAS1*) encoding a hypoxia-inducible factor 2 alpha (HIF2α) double variant with threonine (T) at position 606 and serine (S) at 610. This HIF2α T606/S610 variant was proposed to have a dominant gain-of-function activity ^8^. Subsequent whole genome sequencing analysis of *EPAS1* in 19 breeds of U.S. cattle identified four additional HIF2α variants encoded by *EPAS1* (E270Q, P362L, A671G, and L701F) ^9^. Together, these six amino acid variants comprised eight distinct polypeptide HIF2α sequences. A rooted phylogenetic tree of these HIF2α protein sequences provided a framework for evaluating their potential impact on BCHF in U.S. cattle ^9^. In the present report, our aim was to evaluate *EPAS1* haplotypes encoding HIF2α protein variants for association with BCHF in feedlot cattle, raised at moderate altitudes. Here we show that the HIF2α T606/S610 variant encoded by *EPAS1* was not associated with BCHF in feedlot cattle maintained at 1,200 m in the geographic region experiencing outbreaks. The results are important for understanding the disease mechanism and for future selection of breeding animals with reduced risk for BCHF.

## Methods

### Ethical statement

The experimental design and procedures used during this research project were approved by the Institutional Animal Care and Use Committee (IACUC) of the University of Nebraska-Lincoln (UNL) Experimental Outline Number 139. The UNL animal care program is accredited by the Association for Assessment and Accreditation of Laboratory Animal Care, registered with the United States Department of Agriculture, and assured by the National Institutes of Health Public Health Service Policy on Humane Care and Use of Laboratory Animals. The university meets these goals through review and approval by the IACUC before projects are initiated for any research and educational activities involving vertebrate animals, to assure compliance with all laws, regulations and rules governing the care and use of animals, and by continuing review and monitoring of approved studies. 

No animals were housed at research facilities or cared for by researchers during this study. All animals were privately owned and located at commercial feedlot operations and managed according to their standard operating procedures (SOP), which includes euthanasia of terminal heart failure cases as recommended by the American Veterinary Medical Association ( AVMA). All pen matched unaffected (control) animals were briefly sampled by collecting an ear notch and blood sample and returned to their pens without further involvement in the study. In every instance, efforts were made to ameliorate animal suffering for this incurable, untreatable congesting heart disease.

### Animals and study design

Paired samples from 102 affected calves and their 102 unaffected matched penmate controls were collected from four private commercial feedlots during a 16-month period spanning January 2017 to April 2018. A sample size of 100 matched pairs was targeted based on the frequency of the *EPAS1* T606/S610 variant in Angus cattle (0.22) ^9^. Together with Hardy-Weinberg assumptions, the proportion of discordant pairs having one or two copies of the dominant risk factor was expected to be 0.476 and achieve greater than 80% power to detect an odds ratio of 2.5 using a two-sided McNemar test with a significance level of 0.05 (PASS 2019 Power Analysis and Sample Size Software, version 19.0.2 (NCSS, LLC. Kaysville, Utah, USA).

Potential clinical cases were identified by experienced animal caretakers on horseback (pen riders) and segregated for treatment by moving to a hospital pen. This daily activity is part of the SOP for pen riders, i.e., to identify animals with any potentially serious health problems and move them to a dedicated treatment area. Terminal cases were euthanized by feedlot personnel based on clinical presentation. Euthanasia was accomplished with a well-placed bullet by trained feedlot personnel according to AVMA approved protocols, as part of the feedlot operation’s SOP for terminally ill animals. The case definition included two or more clinical signs specific to BCHF: ventral and intermandibular edema (‘brisket’ and ‘bottle jaw’), jugular vein distention and pulsation, ascites/abdominal swelling, and exophthalmia (‘bug-eye’) ^10, 11^. The case definition also included two or more non-specific clinical signs: dyspnea, abducted elbows, depression, drooped ears, intermittent watery orange diarrhea, tachycardia, exercise intolerance, open mouth breathing, and weight loss. Clinical signs increased with disease progression and in some cases, animals died naturally within 24 hours of clinical presentation, while other cases progressed over a period of days to weeks, and thus provided a window of opportunity to collect fresh samples immediately after euthanasia. Researchers were contacted by the feedlot operator when an animal became ill and needed to be euthanized. Researchers then travelled to the feedlot for sample collection. A presumptive diagnosis based on heart morphology and gross lesions was made at necropsy by animal caretakers (cases one to nine) and veterinarians (cases 10 to 102). Carcasses were enrolled in the study only if there was a postmortem presumptive diagnosis of congestive heart failure at necropsy.

Control tissue samples were collected from non-affected penmates matched for source, arrival date, gender, and breed type (based on source, coat color, horned/polled status, ear size and dewlap). The pen sizes ranged from 100 to 300 animals, and control animals were selected based on their willingness to be driven through the gate by riders on horseback. In two instances, the same control animal was inadvertently used as a penmate match for two clinical cases. None of the control animals developed clinical BCHF signs prior to harvest according to feedlot records and pen rider observations.

Tissue samples for genomic DNA isolation included V-shaped ear notches and EDTA whole blood collected by venipuncture. Squeeze chute devices at the feedlot facilities were used for sample collection with animals that were able to safely enter and exit the gates. Some affected animals were not able to safely enter the device and thus, their samples were collected immediately after euthanasia. The ear tissue was desiccated with granular NaCl in the field and stored at -20°C upon return to the research facility (four to twelve hours later). The plasma and cellular fractions of EDTA whole blood were separated in the field by centrifugation for 10 minutes at 1,350 x g, placed in liquid nitrogen immediately and stored at -80°C upon return. Hearts, livers, blood, and ear notches from unaffected cattle were collected during federally-inspected beef processing at the U.S. Meat Animal Research Center abattoir from six purebred Angus heifers raised and fattened at 578 m and 15 purebred Angus cows maintained for breeding at 578 m.

### DNA extraction and single nucleotide polymorphism (SNP) genotyping

Unless otherwise indicated, reagents were molecular-biology grade. DNA from ear notches was extracted by standard procedures ^12^. Briefly, hair follicles were shaved from the tissue and approximately one half of the ear notch was minced and suspended in 2.5 mL of a lysis solution containing 10 mM TrisCl, 400 mM NaCl, 2 mM EDTA, 1% wt/vol sodium dodecyl sulfate, RNase A (250 ug/ml; Sigma-Aldrich, St. Louis, Missouri, USA), pH 8.0. The solution was incubated at 55°C with gentle agitation. After one hour, 1 mg proteinase K was added (Sigma-Aldrich) and the solution was incubated overnight at 55°C with continued agitation. The solution was transferred to a 15 ml tube containing 2 ml of a phase-separation gel (high-vacuum grease, Dow Corning Corporation, Midland, Michigan, USA) and extracted twice with one volume of phenol:chloroform:isoamyl alcohol (25:24:1), and once with one volume of chloroform before precipitation with two volumes of 100% ethanol. The precipitated DNA was washed once in 70% ethanol, briefly air dried, and dissolved in a solution of 10 mM TrisCl, 1 mM EDTA (TE, pH 8.0). A single multiplex matrix-assisted laser desorption/ionization time-of-flight mass spectrometry (MALDI-TOF MS) assay was used for the six *EPAS1* missense SNPs, as previously described ^9^. Assay design and genotyping was performed at Neogen GeneSeek Operations (Lincoln, Nebraska, USA). The iPLEX Gold technology and MassARRAY DNA analysis system with MALDI-TOF MS (Agena Biosciences Inc., San Diego, California) was used according to the manufacturer’s protocol. Briefly, genomic DNA was amplified in microtiter plates using the supplied reagents. After the PCR, excess nucleotides were dephosphorylated by shrimp alkaline phosphatase. This was followed by a single base extension reaction in which a mix of oligonucleotide extension primers was added together with an extension enzyme and mass-modified dideoxynucleotide terminators. The extension primers are designed to anneal directly adjacent to each SNP site and were extended and terminated by a single complementary base. The extension products were desalted and transferred from the microtiter plate onto a chip array, where they crystalize with a pre-spotted MALDI matrix. The chip array was loaded into the mass spectrometer, where the analyte crystals were irradiated by a laser, inducing desorption and ionization. The positively charged molecules accelerate into a flight tube towards a detector. Separation occurs by time-of-flight, which is proportional to the mass of the individual molecules. After each laser pulse, the detector records the relative time of flight for each extension product and the results were displayed on the machine. Genotypes were scored automatically and summary reports were generated.

### Assigning haplotype phase and statistical analyses

As previously reported, a maximum parsimony phylogenetic tree was used to unambiguously phase protein variants encoded by *EPAS1* haplotypes ^9^. Haplotype-phased protein variants were unambiguously assigned in individuals that were either: 1) homozygous for all six variant sites, or 2) had exactly one heterozygous variant site. The phylogenetic tree was also important for providing a framework for chi-squared testing. The association of *EPAS1* haplotype combinations (diplotypes) with clinical disease was evaluated with two tests. The first was a Pearson’s chi-squared test since it met requirements for appropriate use: nominal categorical data, large sample size (n = 204), and independence of observation assumption (cases were mutually exclusive of controls). There was a small deviation of the assumption that each subject contributes to one and only one cell, because two of the control animals were each inadvertently drawn twice from their pen. None of the animals classified as controls developed clinical disease prior to harvest. A 2 x 4 contingency table was used to test the four diplotypes combinations represented by at least ten animals ^13^. The second evaluation for association of the *EPAS1* T610/S610 variant with clinical disease was McNemar’s test for correlated proportions ^14^. This is the most appropriate test for paired nominal data.

## Results

**Figure 1.**
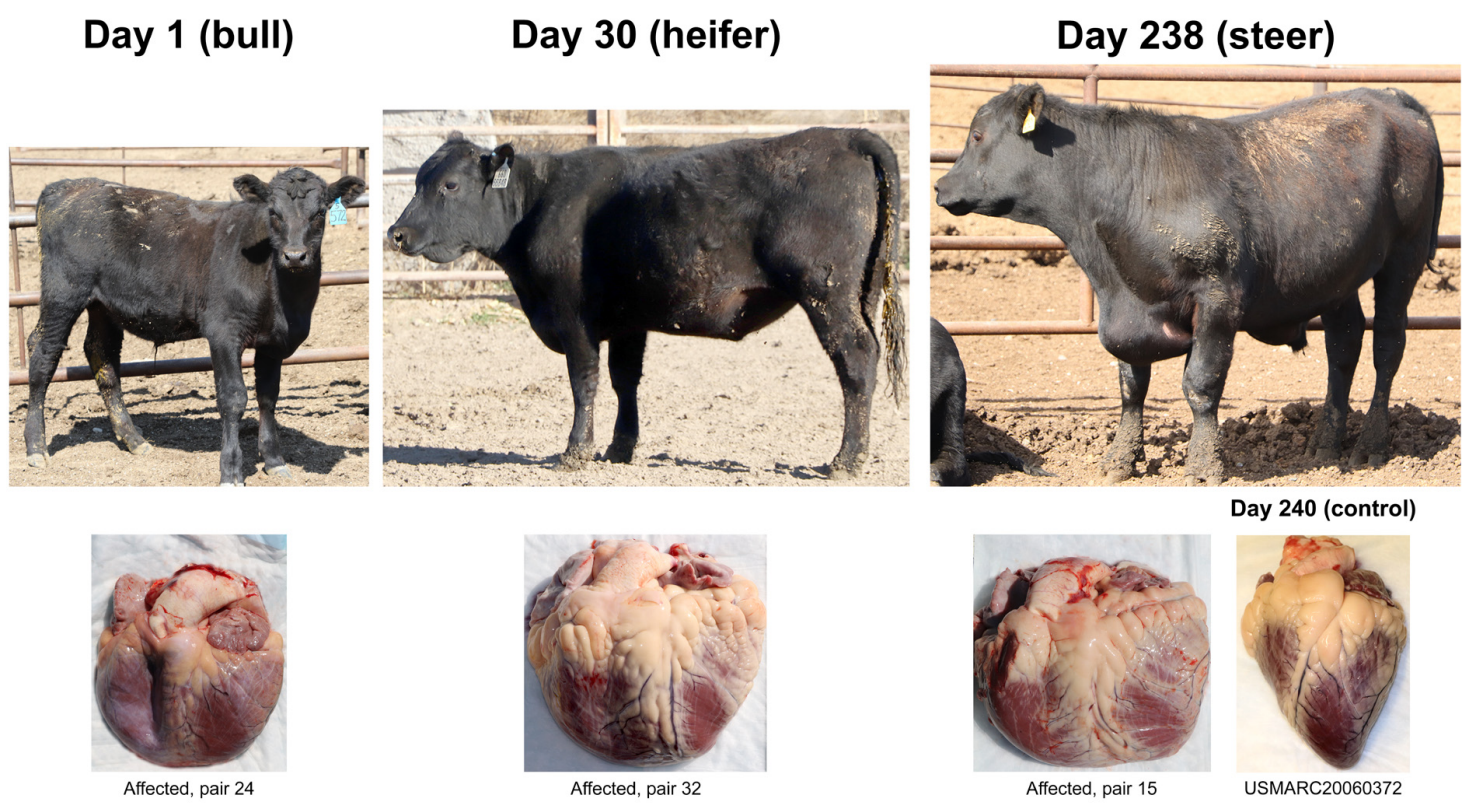
Bovine congestive heart failure (BCHF) clinical cases at different stages of fattening. Three types of animals identified by feedlot pen riders as end-stage heart failure candidates. Clinical cases were born and raised at 1,000 to 1,200 m prior to feedlot arrival. Their respective heart gross morphologies with enlarged right ventricles and pulmonary arteries are shown below each case. The control heart is from an Angus heifer fattened at 550 m.

**Figure 2.**
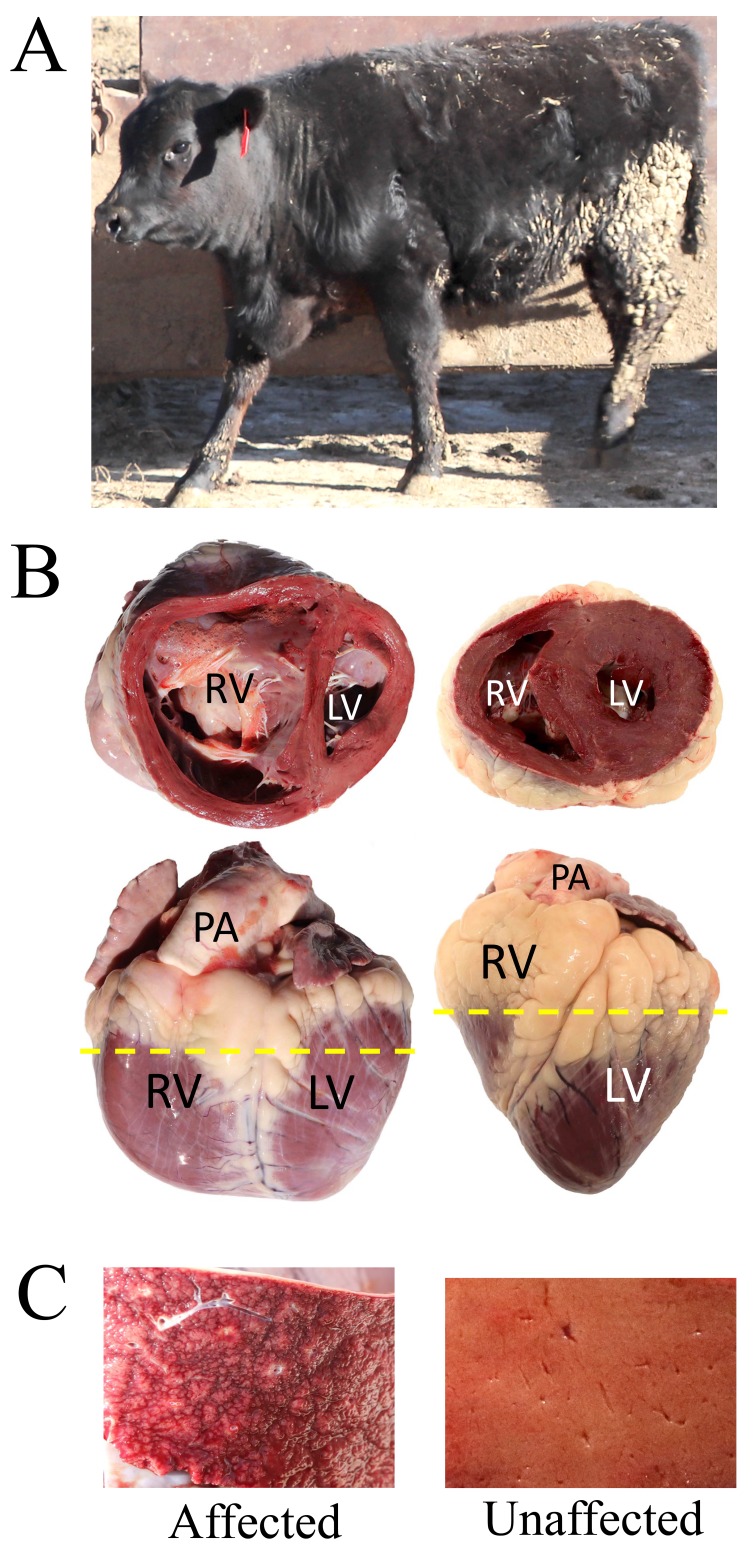
Gross morphological differences of a heart and liver in a representative bovine congestive heart failure (BCHF) clinical case. Panel **A**, affected heifer from (pair 77, Table S1) fattened at 1,200 m. Panel **B**, Gross heart morphology of affected heifer from pair 77 (left) and an unaffected Angus cow maintained for breeding at 578 m (right). The dashed line denotes where each heart was sectioned to show the dorsal half. Abbreviations: RV, right ventricle; LV, left ventricle; PA, pulmonary artery. Panel **C**, cross-section of the respective livers.

The 102 clinical BCHF cases and their penmate controls originated from 30 different sources, with the largest single source contributing 32 matched pairs ( Table 1 and S1, see *Underlying data*). Although breed information was not available for many animals, the pairs were comprised of 100% polled, 93% solid black, and 70% male calves (Table S1, see *Underlying data*). Most of the 102 calves were from known, well-managed herds that focus on using Angus genetics. Clinical cases of BCHF were documented throughout all stages of calf fattening regimens ( Figure 1). The most consistent signs at necropsy included: ventral and intermandibular edema, dilated pulmonary artery and right ventricle, and chronic passive congestion of the liver ( Figure 2). The success of identifying BCHF cases was entirely dependent on highly trained, experienced pen riders. The false positive rate for pen rider-identified BCHF cases confirmed at necropsy was zero at sites NE01, NE04, and WY01; and 50% at WY02 (three cases from six suspected cases). Together, samples from the 102 matched case-control pairs represent a resource for evaluating genetic, biochemical, and physiological questions about the mechanism of BCHF in feedlot cattle.

**Table 1.** Feedlot sites and sources for case-control pairs.

Site ^a^	Altitude m (ft)	Pairs	Sources
NE01	1,242 (4,075)	76	19
NE04	1,163 (3,816)	17	9
WY01	1,263 (4,143)	6	1
WY02	1,280 (4,198)	3	1
	Totals	102	30

^a^NE and WY sites were located in Nebraska and Wyoming, respectively

**Figure 3.**
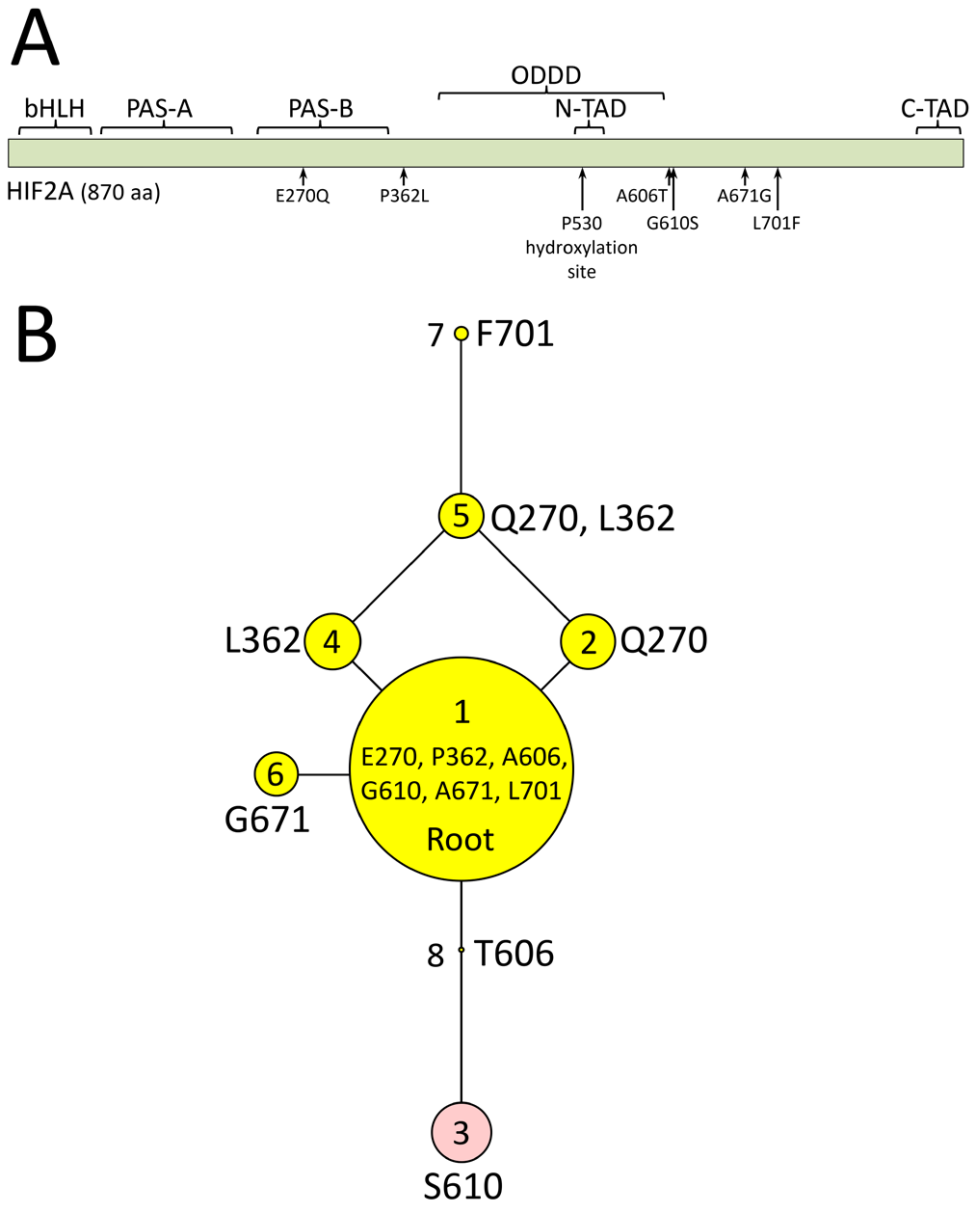
Physical map and rooted maximum parsimony phylogenetic tree of HIF2α protein variants ( *EPAS1* haplotypes). Panel **A**, map of bovine HIF2α domains in relationship to missense mutations found in cattle: bHLH, basic helix-loop-helix domain; PAS-A and PAS-B, Per-Arnt-Sim domains; ODDD, oxygen-dependent degradation domain; N-TAD, N-terminal transactivation domain; C-TAD, C-terminal transactivation domain. Panel **B**, HIF2α polypeptide sequences encoded by haplotype variants. The areas of the circles are proportional to the variant frequency in a group of 1,250 cattle from 46 breeds with each node in the tree representing a different polypeptide sequence of HIF2α that varies by one amino acid compared to adjacent nodes. Reproduced from Heaton *et al.*, 2016 ^9^.

DNA samples from cases and controls were tested for association with known *EPAS1* haplotypes according to the phylogenetic framework presented in Figure 3. Haplotype-phased protein variants (diplotypes) were unambiguously assigned for 98.5% of the animals (201/204, Table S1, see *Underlying data*). The frequency of the common, ancestral *EPAS1* haplotype (‘1’) was nearly identical in the cases and controls (0.67). The T606/S610 variant reported to be dominantly associated with high-altitude pulmonary hypertension (haplotype ‘3’) was less frequent in the cases (0.21) than the controls (0.25, Table 2). In addition, there was no statistical difference between four common diplotypes among the cases and controls in a Pearson’s chi-square analysis ( Table 3). However, the appropriate statistical test for paired nominal data is McNemar's test. This test with 45 discordant pairs showed the T606/S610 variant was not associated with BCHF ( Table 4, OR = 0.73, *p*-value = 0.37, CI _95_ 0.38 -1.4). With 0.44 discordant pairs having the T606/S610 variant, the sample size of 102 pairs achieved 80% power to detect an odds ratio of 2.5 using a two-sided McNemar test with a significance level of 0.05. Frequencies of three other *EPAS1* haplotype variants (Q270, L362, and G671) were less than 0.05 and too rare to analyze with a McNemar test. Thus, an association with *EPAS1* haplotype variants was not detected with BCHF in these cattle.

**Table 2.** *EPAS1* haplotype frequencies among cases and controls.

		Allele frequencies	
Haplotype code	Distinguishing Feature	Cases	Controls
1	Ancestral	0.667	0.672
2	Q270	0.059	0.034
3	T606, S610	0.206	0.250
4	L362	- ^a^	-
5	Q270, L362	0.005	0.020
6	G671	0.064	0.025
7	F701	-	-

^a^Not detected

**Table 3.** Pearson’s chi-squared analysis of *EPAS1* diplotypes among cases and controls.

	*EPAS1* diplotype ^a^								
	1,1		1,2		1,3		1,6		
Disease status	Obs.	*Χ* ^2^	Obs.	*Χ* ^2^	Obs.	*Χ* ^2^	Obs.	*Χ* ^2^	Totals
Affected	43	0.05	11	0.67	30	0.34	8	1.07	92
Unaffected	45	0.05	6	0.69	36	0.35	3	1.09	90
Totals ^b^	88		17		66		11		182

^a^Diplotypes not shown had less than ten total observations and thus were omitted from the analysis.

^b^The chi-square statistic was 4.3 and the *p*-value was 0.23.

**Table 4.** McNemar’s test with *EPAS1* T606/S610 and Q270 variants.

	Risk factor present ^a^	
Matched pairs ^b^	T606/S610	Q270
Case (1), Control (0)	19 pairs	10 pairs
Case (0), Control (1)	26 pairs	5 pairs
Odds ratio	0.73	2.0
*p*-value	0.37 ^c^	0.30 ^c^
95% CI	0.38 - 1.4	0.62 - 7.4

^a^The risk factor was defined as having one or two copies of the *EPAS1* variant.

^b^The ‘(1)’ and ‘(0)’ indicate the presence or absence of the risk factor, respectively.

^c^The *p*-value is the probability of observing this distribution of discordant pairs if there was no association between risk factor and disease.

## Discussion

The present report describes a set of 102 clinical cases of BCHF and their matched controls collected from U.S. feedlots where outbreaks were severe and ongoing. Genetic analyses of *EPAS1* variants did not show an association with BCHF. This result was significant because cases were closely matched with unaffected penmate controls from the same source, sex, breed type, arrival date, and management conditions. In some pairs, ear tag information suggested that animals shared the same sire. Case-control studies are a common and efficient means of studying diseases with a low prevalence and the McNemar's test is the appropriate statistical test for use on paired nominal data ^15^. A major advantage of the present design was the ability to increase the frequency of a potential genetic risk factor with relatively few participants (102 cases), while still evaluating more than 10,000 animals per feedlot site for disease. Nevertheless, results presented here suggest it is unlikely that genetic variation at the *EPAS1* locus was a significant genetic risk factor for BCHF in these cattle.

The lack of *EPAS1* association with BCHF is inconsistent with that reported in 2015 by Newman *et al.* for cattle with high-altitude pulmonary hypertension ^8^. However, there were important differences in the cattle types, age, environment, and clinical definitions between the two studies. Animals in the 2015 study were mature, pastured cattle, maintained at 1,478 to 2,618 m, and had pulmonary arterial hypertension, as measured by heart catheterization. In the present study, animals were yearling steers and heifers, raised and fattened at approximately 1,200 m, and had end-stage heart failure. Thus, it is possible that the animals in these respective studies are suffering from similar but different diseases. Another possibility is the cattle described by Newman *et al.* were affected by a right-sided ventricular heart failure initiated by hypobaric hypoxia, whereas the cattle in the present study had a left-sided ventricular failure, resulting in tissue hypoxia and subsequent BCHF. The latter was reported in 2019 in cattle fattened at 1,200 m suffering end-stage heart failure ^6^. However, it is unknown whether the disease pathogenesis was a distinguishing factor in these studies. One remarkable feature of the BCHF clinical cases in the present study were those displaying signs of the disease at feedlot arrival and soon after. This is consistent with the hypothesis that fattening is not the underlying cause of disease in these cattle.

## Conclusions

Protein variants encoded by *EPAS1* haplotypes were not significantly associated with BCHF in fattened cattle at moderate altitudes in the North American Western Plains. Thus, identifying the genetic risk factors underlying this form of the disease may require a wider search.

## Data availability

### Underlying data

Figshare: Table S1. Metadata and phased *EPAS1* diplotypes for 102 case-control pairs. https://doi.org/10.6084/m9.figshare.8862089.v1 ^16^

### Extended data

Figshare: Table S2. Summary of 36 possible *EPAS1* diplotypes and their frequency in cases and controls. https://doi.org/10.6084/m9.figshare.8862152.v1 ^17^

Figshare: Table S3. *EPAS1* haplotype frequencies in cases and controls. https://doi.org/10.6084/m9.figshare.8862179.v1 ^18^

Figshare: Table S4. *EPAS1* risk factor scoring used in McNemar’s Test with 102 matched pairs. https://doi.org/10.6084/m9.figshare.8862200.v1 ^19^

Data are available under the terms of the Creative Commons Zero "No rights reserved" data waiver (CC0 1.0 Public domain dedication).
