# Supplementary material for: Evaluation of EPAS1 variants for association with bovine congestive heart failure
Source: F1000Res. 2019 Jul 25;8:1189. [Version 1] doi: 10.12688/f1000research.19951.1 (PMC6733380; doi:10.12688/f1000research.19951.1)
Supplement: Peer review history

# Peer review: Reviewer response for version 1

**DOI:** 10.5256/f1000research.21898.r52934

**Published:** 2019-09-13

**Authors:** Franklyn B. Garry

**Affiliations:** 1 Integrated Livestock Management, College of Veterinary Medicine and Biomedical Sciences, Colorado State University, Fort Collins, CO, USA

- This is an excellent report that investigated whether a specific genetic factor is involved in the development of an increasingly important disease of feedlot animals managed at moderate altitudes. It is scientifically sound and well written. The disease condition is Bovine congestive heart failure, which is clinically similar to altitude-induced pulmonary hypertension with cor pulmonale and right heart failure, commonly known as High Altitude disease or Brisket disease. A previous investigation suggested that a variant of EPAS1 was strongly associated with the development of Brisket disease. This investigation explored whether that gene was associated with BCHF. 
- This was a case control study that was well designed and described. The explanation of experimental and statistical methods and results was very clear. 
- This is an important area of inquiry because BCHF is an important disease problem with significant impact on feedlot producers, for which there are currently no good preventive, management or treatment practices. Previous work has shown that there are significant differences in the pathologic findings of animals affected by BCHF versus Brisket disease, and this project shows that the genetic basis of the two problems is also likely to be different.  
- The authors describe that tissues were collected from some unaffected animals at slaughter, but there was no description of what analyses were performed on these tissues. I am assuming these were used to assess gross morphologic changes in comparison to the tissues of affected animals at necropsy, but this was not described. 
- There is a typographical error in the last sentence of the second paragraph of the Methods section, written as 'congesting' rather than 'congestive'. 

I confirm that I have read this submission and believe that I have an appropriate level of expertise to confirm that it is of an acceptable scientific standard.

# Peer review: Reviewer response for version 1

**DOI:** 10.5256/f1000research.21898.r52931

**Published:** 2019-09-03

**Authors:** Emily L. Clark

**Affiliations:** 1 The Roslin Institute, Royal (Dick) School of Veterinary Studies, University of Edinburgh, Midlothian, UK

- The aim of the study described in this manuscript was to evaluate a known genetic risk factor for BCHF in feedlot cattle raised at moderate altitudes by screening of *EPAS1 *haplotypes in paired cases and controls.
- BCHF is a costly health problem for cattle producers in the Western Plains with losses exceeding $250,000 annually and a 11 in 10,000 animal mortality rate. Consequently, reducing the impact of BCHF is a high priority. A potential genetic risk factor for BCHF was reported in 2015 for cattle with high-altitude pulmonary hypertension (1,478 to 2,618 m) but it was unclear at the outset of this study whether this genetic risk was also prevalent in feed-lot cattle at moderate altitudes. 
- The authors indicate that "Affected cattle were typically bred and managed with the aim of achieving high carcass quality." Presumably this could indicate there is some pleiotrophic effect of a gene influencing both traits and/or other concomitant traits? Or at least breeding for high carcass quality or other traits might be antagonistic to BCHF prevalence? As such measuring gene expression in lung, liver and heart in affected and unaffected animals in future studies may yield additional information particularly in the pathways that are involved in the disease; I assume the authors might already be working on this. 
- The experimental design is clear but defining “pen-matched” and “pen mate” unaffected controls at the first mention in the animal and study design would be helpful to the reader. 
- When the authors indicate that when an animal died naturally this “provided a window of opportunity to collect fresh samples immediately after euthanasia.”, could they provide details of which tissues they collected? Were tissues collected for RNA as well as DNA for genotyping? Presumably samples were also collected for pathology but this isn’t mentioned here.
- Similarly for “Control tissue samples were collected from non-affected penmates”, was this just ear tissue as mentioned in the methodology or other tissues, which could be used for RNA-Seq and gross pathology/histology?
- “Hearts, livers, blood, and ear notches from unaffected cattle were collected during federally-inspected beef processing at the U.S. Meat Animal Research Center abattoir from six purebred Angus heifers raised and fattened at 578 m and 15 purebred Angus cows maintained for breeding at 578 m.” It needs to be clear from this sentence that these were for visual and gross morphological comparison with affected BCHF organs, as this only becomes obvious in Figure 1.
- Similarly in figure 2 it is clear that heart and liver were collected to assess gross morphological differences between affected and unaffected animals but this isn’t clear from the animal and study design section. 
- As a general comment to summarise the above comments relating to the sample collection could the authors clarify in the animal and study design section which tissues were collected and from which groups of animals in the text, and provide additional details in the form of a table in "underlying data" if necessary? This is particularly important because as the authors suggest the samples and information from these cattle are themselves a very useful resource for studying the disease, and knowing exactly which samples have been collected might reduce the chance of duplicated effort in the future.
- Technically I believe the results section of this manuscript is sound. The methodology for genotyping using a custom iPLEX gold genotyping array for the six EPAS1 missense SNPs has been evaluated in previous studies by the authors and to the best of my knowledge the statistical tests used to analyse the results have been interpreted accurately. 
- In the discussion the authors mention “In some pairs, ear tag information suggested that animals shared the same sire.” What was the proportion of the total number of animals that shared the same sire? Is it possible to determine how many different sires produced the 102 cases and matched pairs? Were any animals included in the study twins? I am not sure of the twinning rate in these cattle, I assume it might be quite low, but are there ever cases where one twin is affected by BCHF and the other unaffected?
- The lack of an association with the *EPAS1 *haplotype variants and BCHF, in the feed lot cattle raised at moderate altitude included in this study, is a negative but nevertheless important result, that will be informative to producers, given the importance of BCHF. 
- This is a well-designed and executed study that has been presented clearly and informatively by the authors.

I confirm that I have read this submission and believe that I have an appropriate level of expertise to confirm that it is of an acceptable scientific standard.

# Peer review: Reviewer response for version 1

**DOI:** 10.5256/f1000research.21898.r52928

**Published:** 2019-08-27

**Authors:** Donagh P. Berry

**Affiliations:** 1 Animal and Grassland Research and Innovation Centre, Teagasc, Fermoy, Ireland

The report is extremely well written and of high quality; crucially important that "negative results" are still published. In the abstract please clarify that the n=102 is 102 pairs - it a bit ambiguous. it would have been nice to genotype the animals on some for of SNP chip to determine the breed composition (you allude to not knowing) and also the pedigree structure (may influence your analysis). Other than for breeding purposes (end of the introduction) the results of such an approach can be used for personalised management (including purchasing). please re-write the 7th line of the second paragraph in the M&M; i can't understand what is being said - maybe there is a missing word or two

I confirm that I have read this submission and believe that I have an appropriate level of expertise to confirm that it is of an acceptable scientific standard.
